# Use of variable online visual feedback to optimize sensorimotor coding and learning of a motor sequence

**DOI:** 10.1371/journal.pone.0294138

**Published:** 2023-11-27

**Authors:** Marie Bernardo, Yannick Blandin, Géry Casiez, Cécile R. Scotto

**Affiliations:** 1 Centre de Recherche sur la Cognition et l’Apprentissage, Université de Poitiers, Université de Tours, CNRS, Poitiers, France; 2 Université de Lille, CNRS, Inria, Centrale Lille, UMR 9189 CRIStAL, Lille, France; 3 Institut Universitaire de France (IUF), Paris, France; University of Turin, ITALY

## Abstract

The present study characterized the impact of reliable and/or unreliable online visual feedback and their order of presentation on the coding and learning of a motor sequence. Participants practiced a 12-element motor sequence 200 times. During this acquisition phase, two groups received a single type (i.e., either reliable or unreliable) of online visual feedback, two other groups encountered both types of feedback: either reliable first then unreliable, or unreliable first then reliable. Delayed retention tests and intermanual transfer tests (visuospatial and motor) were administered 24 hours later. Results showed that varying the reliability of online visual information during the acquisition phase allowed participants to use different task coding modalities without damaging their long-term sequence learning. Moreover, starting with reliable visual feedback, replaced halfway through with unreliable feedback promoted motor coding, which is seldom observed. This optimization of motor coding opens up interesting perspectives, as it is known to promote better learning of motor sequences.

## Introduction

Multisensory integration is the best way of achieve longlasting learning, as it allows learners to quickly adapt their behaviors to changes in the environment (see [1], for a review). However, in visuomanual tasks, individuals often rely on visual feedback [2]. As a consequence, their learning is based much more on allocentric representations (i.e., associated with visuospatial coding in extrinsic object-centered coordinates) than on egocentric ones (i.e., associated with motor coding in intrinsic body-related coordinates) [3–6], which are often found to be fairly underdeveloped [7]. Sensorimotor coding has been found to be influenced by the reliability of visual feedback [8,9], and the variability of practice (see [10], for a review). However, there does not appear to have been any research on the combined effects of these two factors on sequential tasks. In the present study, we therefore sought to pinpoint the impact of precise and/or variable types of online visual feedback and their order of presentation on the coding and learning of a motor sequence.

When practicing a motor task in a given environment, individuals quickly identify the sensory source that is most likely to ensure maximum success on that task (*specificity of practice hypothesis*; [2]), to the detriment of the other sources. For visuomanual tasks, which require great visuospatial accuracy, visual feedback has been identified as the dominant sensory source [2,11] (see [12] for a review). However, if this information subsequently becomes unavailable, movement planning and control will be disrupted, resulting in major aiming errors. A similar form of dominance can be observed for cognitive representations of tasks. The manner in which we code, process and transfer a movement sequence has been extensively investigated (see [12], for a review) since Lashley’s groundbreaking study about serial behavior [13]. The author thus proposed that sequential behaviors (e.g., reaching, grasping, locomotion; or in another register: language and logical reasoning) are hierarchically organized according plans (see also [14]).

Many authors agree that motor skills are coded in two distinct and independent representational systems that contribute to specific learning and transfer abilities [3,4,15–18]. Whereas visually acquired information about stimuli and effector locations is initially coded in extrinsic object-centered (i.e., visuospatial) coordinates, patterns of muscle activation are coded in intrinsic body-related (i.e., motor) coordinates [3–6]. Thus, intermanual transfer tasks are characterized by the dominance of visuospatial coding (e.g., [6,7,19–21]) and relative underuse of motor coding (e.g., [7,19,21]). During these transfer tasks, participants perform the sequence with their nondominant hand and either the same visuospatial mapping (i.e., visuospatial transfer, TVS) or a mirror sequence to maintain the motor mapping (i.e., motor transfer, TM) (see Task, Groups, and Procedures for details). As performance on a task featuring changes in either sensory information or context depends on the ability to activate either visuospatial or motor coding (see [1] for a review), it is important to promote both (i.e., mixed coding), especially motor coding [7].

One way to promote motor coding is to manipulate the reliability of online visual feedback [9,20]. Bernardo et al. [20] recently observed that unreliable online visual feedback (i.e., large point cloud cursor) promoted both visuospatial and motor coding, whereas reliable feedback (i.e., single dot cursor) promoted accurate visuospatial coding to the detriment of motor coding. The theory behind this is that when online visual feedback is unreliable, learners turn to other sensory sources [8,9,21], as predicted by the Bayesian approach to multisensory perception [22]. Bernardo et al. [20] suggested that in a sequential task, unreliable online visual feedback pushes individuals to engage earlier and to a greater extent in the processing of proprioceptive information, but without reaching the level of long-term retention achieved by individuals who are given reliable feedback.

Another probable way of promoting motor coding is to introduce variability into the practice (e.g., [8,9,23–26]). In concrete terms, introducing variability in the acquisition phase involves either modifying the conditions in which the movement has to be carried out or manipulating the characteristics of the movement itself [27–29]. One of the most common paradigms developed to manipulate variability of practice consisted in modifying the order of practice (for reviews, see [10,30,31]). These protocols are based on the switch–more or less frequent–between the variations of the task (i.e., *contextual interference*). However, variability could also be introduced at the feedback level, i.e., during the task. Despite the major role of feedback for learning (see above), this option has been substantially under considered (for review see [10]). No matter how variability is handled, its beneficial effects on learning are intrinsically linked to the notion of generalized motor programs in Schmidt’s schema theory [28,31]: by introducing variability, we increase the reliability of the processes responsible for controlling the movement and expand participants’ repertoire of motor solutions. Thus, in contrast to constant practice, which does not involve any modification in the conditions for carrying out the task during the acquisition phase, variable practice leads to better learning performance [32,33]. This is because it favors the extraction of the abstract rule, allowing for better parametrization of the gestures at recall [34]. Learners are therefore able to adapt their practice to the environmental context (for reviews, see [10,35–37]). As for online visual feedback, introducing variability results in richer and more adaptable representations of the task [25]. To our knowledge, the only attempt to assess benefits of variability of practice *and* sensory feedback on motor learning only referred to the presence or absence of the visual cursor [25] and not its reliability, that mainly modulates learning abilities [8,9,21].

In the present study, we specifically examined the effect of modulating the reliability of online visual feedback during acquisition and the influence of order of presentation (i.e., reliable or unreliable feedback first) on sensorimotor coding and sequence learning. We defined four experimental groups: two with a single type of online visual feedback in the acquisition phase, and two with both types: reliable cursor throughout the acquisition phase (R); unreliable cursor throughout the acquisition phase (U); reliable then unreliable feedback (RU); and unreliable then reliable feedback (UR). We evaluated learning and coding with a task including an acquisition phase, a retention phase (+24 h) and an intermanual transfer phase (+24 h). Retention was specifically used to test the sequence’s long-term retention and intermanual transfers to assess visuospatial (*i*.*e*., TVS) and motor coding (*i*.*e*., TM) of the sequence (see Task, Groups, and Procedures for details). Regarding learning, we hypothesized a deleterious effect of a total lack of reliability (i.e., U) and a minor or even absent effect when strong reliability was temporarily present (i.e., RU and UR) or totally present (i.e., R). In the variable practice groups (i.e., RU and UR), we expected to observe a modification in the coding, as participants’ exposure to both types of online visual feedback would push them to modify their representations of the task. In particular, while the reliable group (i.e., R) should present better performance in visuospatial transfer than in motor transfer, we predicted the opposite for the other three groups. More importantly, as visual feedback has been shown to be crucial for learning onset, we expected the RU group to benefit more from variable practice than the UR group, especially regarding the development of motor coding.

## Method

### Participants

We recruited 80 right-handed adults (mean age = 18.96 years, *SD* = 1.22; 20 women and 49 men) among students at Poitiers University. They received a course credit in exchange for taking part. They each signed an informed consent form prior to the experiment, which was approved by the local ethics committee (no. 201965). All participants stated that they had no history of neurological or sensorimotor disorders and had normal or corrected-to-normal vision. Participants were randomly assigned to one of the four groups: R (reliable cursor), U (unreliable cursor), RU (i.e., reliable dot cursor first, then unreliable point cloud cursor); UR (i.e., unreliable then reliable cursor). Data for the groups R and U were draw from Bernardo et al. [20]. The number of participants assigned to each group was computed using power analysis software (G*Power 3.1; University of Düsseldorf, Germany), based on empirical data yielded by a similar experimental design [20]. From this empirical data, the implemented effect size, we used eta^2^ or *d* (0.84 = large effect size) as a measure of effect size, and Pearson’s *r* (0.5 = moderately strong correlation) to measure linear correlations. The computation was based on a mixed 1 (between-participants factor; 4 groups) x 1 (within-participants factor; 27 conditions) experimental design. Statistical significance was set at *p* < .05, and power was set at 0.95. Results indicated that 8 participants per group would be required for the experiment. However, the subgroups created for the counterbalancing (see “Task, Groups and Procedures” subsection) required the number of participants to be a multiple of 4. There were therefore 20 participants in each group. Eleven participants’ data could not be used: 6 due to technical recording problem and 5 were outliers (median ± 2.5 *SD* [38]). The group R was then composed of 17 participants (2 women, 15 men; mean age = 19.35 years, *SD =* 0.61), 18 for the group U (7 women, 9 men; mean age = 19.31 years, *SD* = 0.48), 19 for the group RU (6 women, 13 men; mean age = 18.95 years, *SD* = 1.72) and 17 for the group UR (5 women, 12 men; mean age = 18.24 years, *SD* = 1.25).

### Apparatus

To administer the task, we used a high-definition screen (Acer ROG PG278QR, 2560 x 1440 pixels, 165 Hz refreshment, connected to an NVIDIA GeForce GTX 1080 graphic card) associated with a Dell computer (Intel(R) Xeon(R) W-2123 CPU @3.60 GHz; Windows 10 professional). We also used a WACOM Intuos4 XL high-definition graphics tablet (1240-d version 2.0, resolution: 5080 lines per inch– 0.005 mm per point, sensitive area: 493 x 304 mm, 200 Hz refreshment). A digital stylus allowed participants to navigate the screen via the tablet. The cursor was only displayed on the screen when the stylus was in contact with the tablet. Data from the stylus were processed by a custom-built application written in C++ using Qt and LibPointing [39]. We used absolute mapping between the tablet and screen, with a gain value of 1 (i.e., what was seen on the screen corresponded to what was done on the tablet). The center of the screen corresponded to the center of the tablet.

### Task, groups, and procedures

The experiment was designed with three phases: an acquisition phase, a retention phase and a transfer test according to Boutin et al. [40]. During the acquisition phase (day 1), participants’ group completed a pointing sequence with different cursor reliability (see below), sequence which was new (N1/N2) or repeated (R0 to R20). Performances were compared with pointing sequences performed on day 2, with the different cursor reliabilities (retention tests). In addition; participants also performed pointing with the left hand to assess generalization of learning (intermanual transfer tests). See below for details of the experimental task and procedure.

Prior to the experiment, participants were asked to adjust the height and position of their seat, so their right hand was at desk height and approximatively in the center of the tablet. The task consisted in making arm movements to point at each of the targets in turn, as they were displayed on the screen in an ordered sequence. Participants were asked to point as fast as possible throughout the trials. Four targets (⌀ = 1 cm) were positioned horizontally on the screen at -21 cm, -7 cm, 7 cm, 21 cm from the center and remain visible throughout the task.

**Fig 1:**
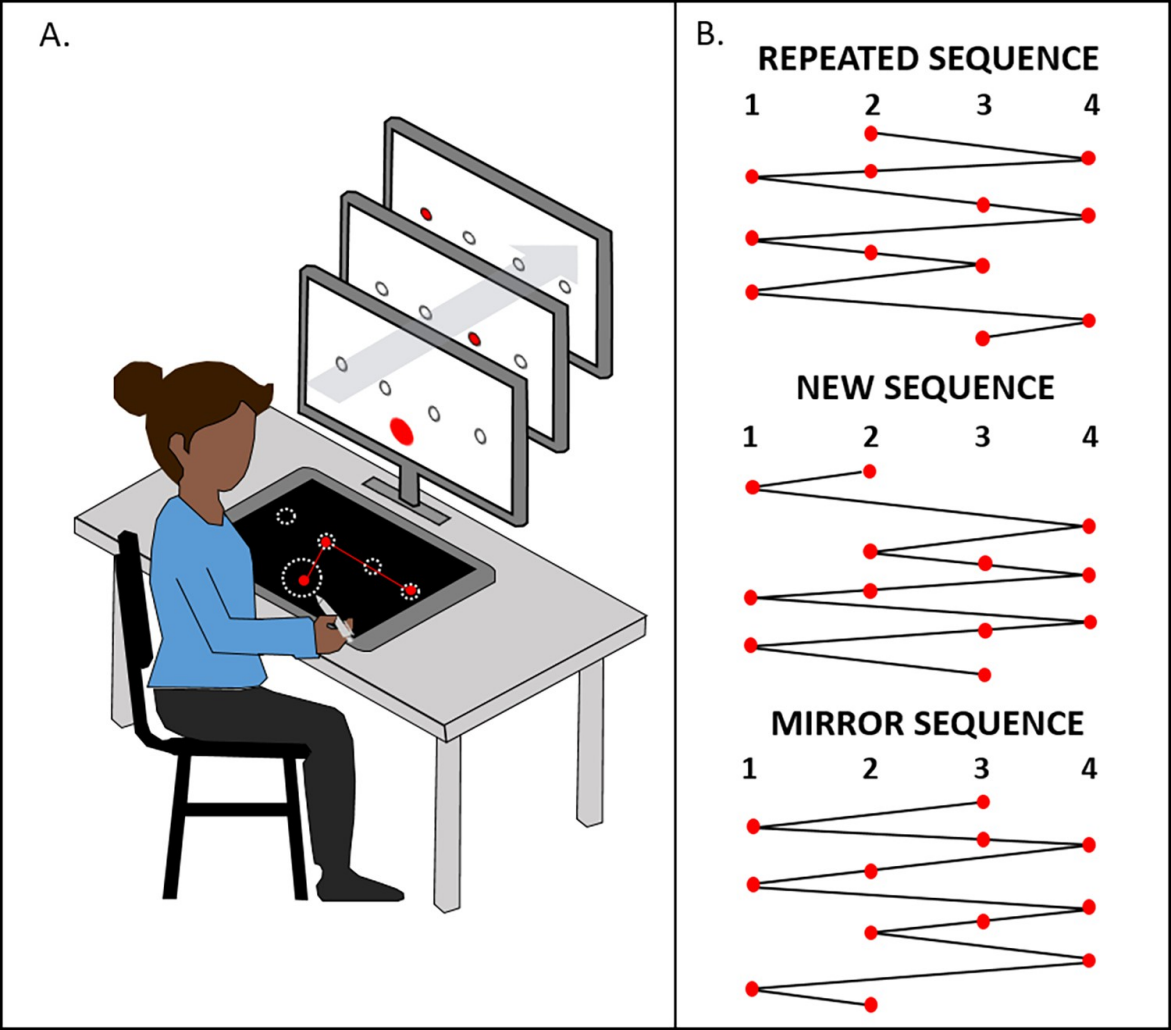
**A**. Set-up. Participants sat in front of a screen and pointed to targets via the tablet. To launch a block of trials, they had to point to a centered calibration target in the bottom middle of the screen. This target then disappeared, and they had to point to each successive target as soon as it lit up (represented here on the three screens), without lifting the stylus from the tablet. The targets and stylus trajectory are depicted on the tablet solely to facilitate understanding; no visual stimuli were displayed on the tablet. **B.** Design of the three sequences. The repeated sequence was the one to learn, and was used in all the repeated blocks (R0-R20), as well as the retention blocks (RETr and RET) and TVS block. The new sequence was different, but had the same characteristics as the repeated sequence, and was used in the pre- (N1) and post- (N2) tests. The mirror sequence (mirror of repeated sequence) was used in the MT block.

To make the procedure easier to understand, we numbered the targets, from left to right (i.e., 1, 2, 3, and 4 ; Fig 1B). When a target was activated (i.e., when participants had to point to it), it turned red. The rest of the time, only the circular outline remained visible. To start each block of trials, participants had to point to a calibration target (⌀ = 6 cm) in the bottom middle of the screen (i.e., Fig 1A), after which it disappeared. Thereafter, the first target in the sequence turned red and participants had to move the stylus across the tablet as quickly as possible, in order to validate the target by crossing its perimeter: they did not have to stop the cursor *in* the target. As soon as the target was reached, it lost its red color and the next target turned red, and so on and so forth, until all 12 elements (i.e., targets) in the sequence had been crossed. This sequence was repeated 10 times in each block. Each time participants finished a block (i.e., 10 x 12 elements = 120 successive targets), they could take a quick break (i.e., at least 5 seconds) before beginning the next block. During the acquisition phase (i.e., R0-R20, N1 and N2), participants knew their movement time at the end of each block so that they could use this feedback to improve their performance. It corresponded to the movement time in seconds for the block performed and was displayed on the screen. Participants were instructed not to lift the stylus from the tablet, otherwise the cursor would disappear. Thus, while the targets were always visible, the aiming cursor which constituted the online visual feedback was only available if the participants left the stylus on the tablet. More details on the cursor are provided in the next paragraph.

Three different sequences were used during the experiment, in accordance with Boutin et al. [40]. Each one comprised 12 elements (Fig 1B). The repeated sequence (targets: 2 4 2 1 3 4 1 2 3 1 4 3) was the sequence to learn. The new sequence (targets: 2 1 4 2 3 4 2 1 4 3 1 3) was an unpracticed sequence with the same characteristics as the repeated sequence to be compared with the repeated sequence (see [40]). Therefore, the new sequence was presented in pre- and posttests. Finally, the mirror sequence (targets: 3 1 3 4 2 1 4 3 2 4 1 2) was used for the TM block.

**Fig 2:**
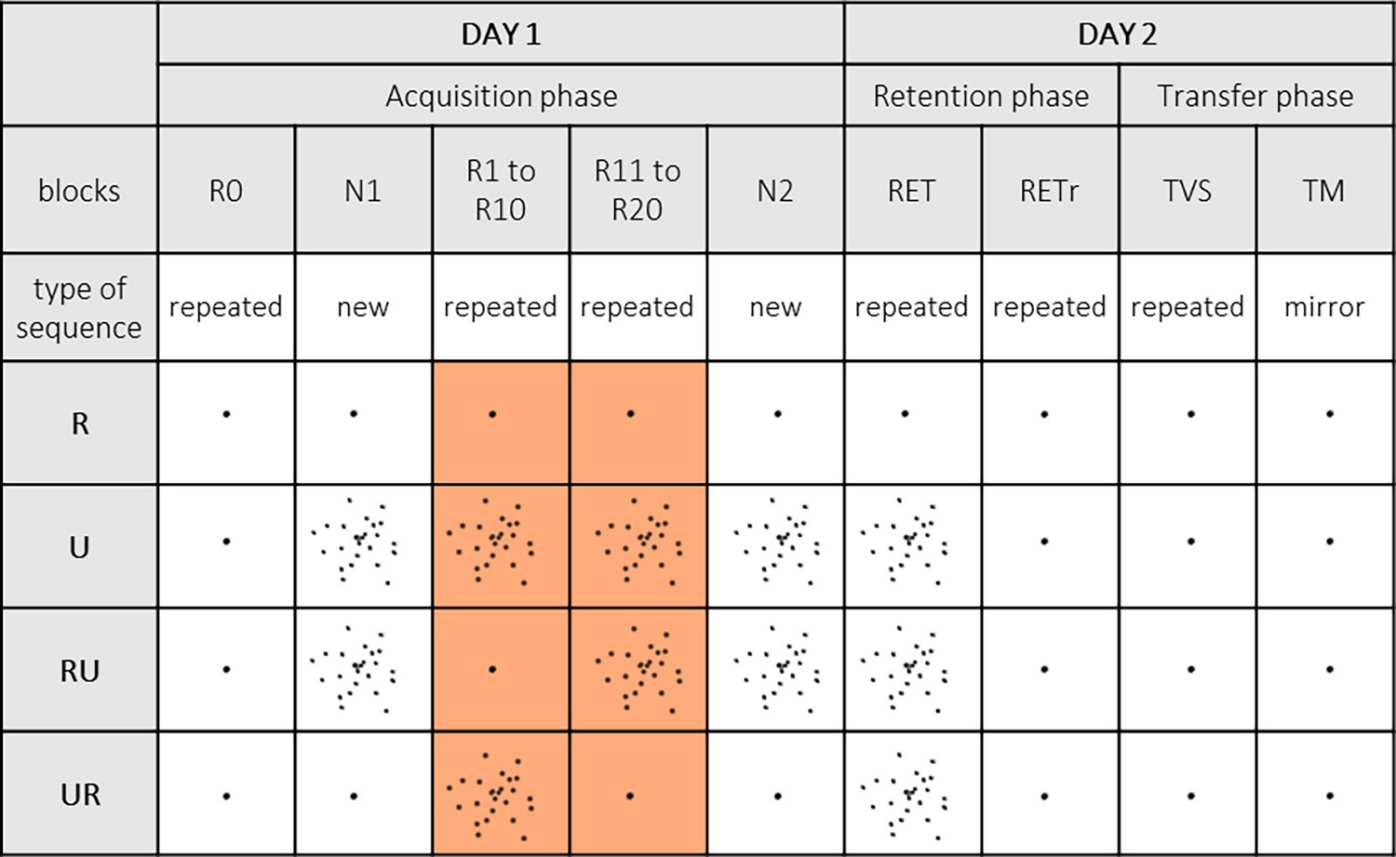
Experimental procedure and cursor use. Experimental design. Four groups performed the experiment over 2 consecutive days, with different levels of cursor reliability during the acquisition phase: R: Group with reliable single dot cursor; U: With a cloud of dots as a cursor; RU: Group with reliable cursor for the first part of the acquisition10 blocks (blocks R1-R10); then unreliable for the last 10 blocks (blocks R11-R20); UR: Group with unreliable cursor for the first part of the acquisition10 blocks (blocks R1-R10); then reliable for the last 10 blocks (blocks R11-R20). Day 1 (acquisition phase): Starting block (R0) with repeated sequence and reliable cursor for all groups. N1 and N2: Pre- and posttest blocks with new sequence and same cursor as R20 for comparison. R1 to R20: Training blocks with the repeated sequence and cursor according to experimental group. Day 2 (retention phase): Retention block with repeated sequence and unreliable cursor for all groups except for R (reliable cursor) (RET); retention block with repeated sequence and reliable cursor for all groups (RETr). Day 2 (transfer phase), TVS: Visuospatial transfer block with repeated sequence, reliable cursor and left hand. TM: Motor transfer block with mirror sequence, reliable cursor and left hand.

Fig 2 summarizes the experimental conditions and procedure. We used two types of cursor to manipulate the reliability of the online visual feedback [20,21]: the reliable cursor was a single black dot (⌀ = 1 mm), while the unreliable cursor corresponded to a sparse cloud made up of 25 black dots (⌀ = 1 mm, transparency = 40%) with a two-dimensional Gaussian distribution (*SD* = 20 mm). Five different clouds with these characteristics were generated and used for each block of 10 repetitions, in a pseudorandom and counterbalanced order. We had defined four groups according to the reliability of the cursor available during the acquisition phase. In the R and U groups, the cursor was the same throughout the acquisition phase: reliable for the first, and unreliable for the second. The other two groups each encountered both types of cursor during the acquisition phase. In the RU group, the cursor was reliable for Blocks R1-R10, then unreliable for Blocks R11-R20. In the UR group, an unreliable cursor was followed by a reliable one.

The procedure was carried out in three phases, spread over 2 consecutive days (Fig 2). The first day, participants performed the acquisition phase. The initial block (i.e., R0) was the same for all four groups, and featured the repeated sequence and the reliable cursor. This first block served to establish the participants’ baseline level, for comparison with their retention performance. Following R0, a block was presented with the *new sequence* (i.e., N1). Participants then performed 20 blocks of the *repeated sequence* (i.e., repeated blocks R1-R20). At the 10^th^ block, participants took a 10-minute break. It was after this break that the cursor switch took place for the RU and UR groups. Following the completion of the subsequent 10 repeated blocks, all participants performed a second block featuring the *new sequence* (i.e., N2). Blocks N1 and N2, respectively completed before and after practice of the repeated sequence, allowed us to measure the sequence-specific learning of the repeated sequence (see [41], for a review; see [19,40] for a similar procedure). Because we compared N1 and N2 with R20, they were completed with the same cursor: reliable cursor for R and UR, and unreliable cursor for U and RU. After an interval of 24 hours, all the participants returned to perform the *retention and transfer tests* to judge the persistence and transfer of learning. One of the retention blocks featuring the repeated sequence (RETr) was performed with the reliable cursor, while the other (RET) was performed with the unreliable cursor, except for participants in R, who had never encountered this cursor. The RETr block was run to provide inter-groups comparison on learning: participants of all groups completed the sequence with the same reliable cursor. RET was completed with the cursor’ reliability available at the end of acquisition (Reliable or Unreliable, depending of groups) to ensure that performance was maintained from the end of acquisition to RET (intra-group comparison). Finally, all the participants performed intermanual transfer tests with the reliable cursor, to judge the generalization of learning [7,8,18,19,40]. More specifically, the transfer tests measured the extent to which the repeated sequence had been stored and coded. Participants performed a VisuoSpatial Transfer block (*i*.*e*., TVS) with the *repeated sequence* and the stylus in the left hand. Therefore, because the spatial mapping of the targets remained identical to the one used with the repeated sequence, an opposed pattern of flexion-extension had to be produced. They also performed a *Motor Transfer* block (*i*.*e*., TM) with the *mirror sequence* (Fig 1B) and the stylus in the left hand. In this test, the sequence was mirrored compared the one used with the repeated sequence but a similar pattern of flexion-extension had to be produced. The order of presentation of the two retention blocks and the two transfer blocks was counterbalanced within each group.

### Data processing

Data processing was performed using MATLAB (version r2020b; Mathworks, Natick, MA). Position data from the tablet were low-pass filtered with a dual-pass, no-lag Butterworth filter (cutoff frequency: 10 Hz; order: 2). These data were used to determine movement time (MT) per block. This corresponded to the mean time (in ms per element) between the validation of the first and last elements in the sequence (i.e., 11 pointing movements). Outlier values in practice trials (median ± 2.5 *SD*) were removed from the analysis [38], representing 3.7% of the data. Data analyses consisted in running mixed analyses of variance (ANOVAs) on MT per element with JASP (version 0.14.1; JASP, 2020). The level of significance was set at .05, and the effect size was reported for all significant effects (eta-squared, η^2^ [42]). When necessary, we applied the Greenhouse-Geisser correction, and reported the corrected degree of freedom. To test our hypotheses, we also ran planned comparisons (indicated in each section of the results).

### Transparency and openness

We reported how we determined our sample size (see “Participants” subsection), all data exclusions, all manipulations, and all measures in the study (see “Data processing” subsection). All data and MATLAB codes are available at [https://osf.io/b2yan/]. The study design and analysis were not preregistered.

## Results

**Fig 3:**
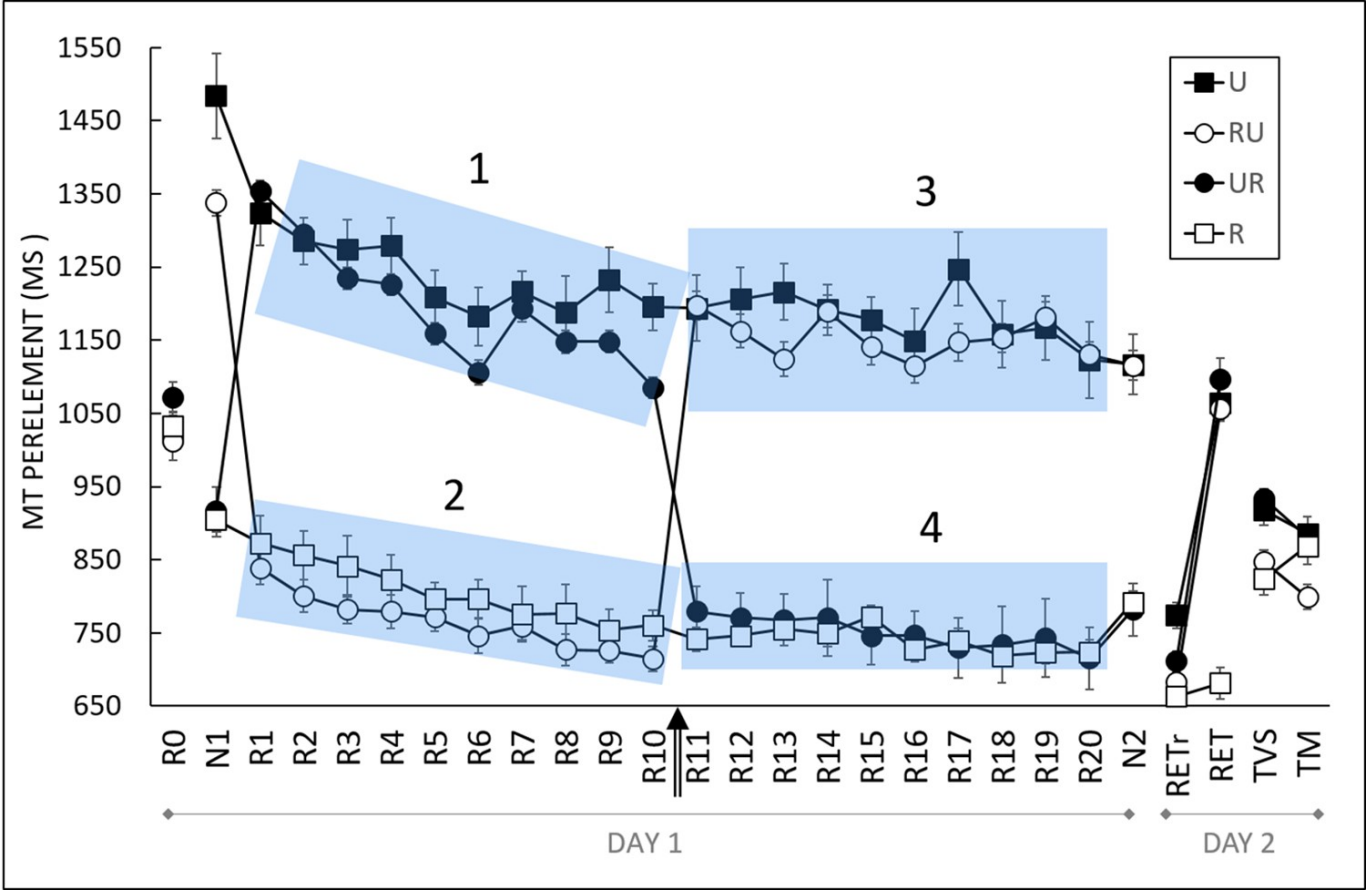
Movement time (MT) per element during acquisition, retention and transfer. R0-R20: repeated blocks. R11: Cursor change block for UR and RU (depicted by the arrow). N1 and N2: New blocks. RET: Retention with unreliable cursor (except for R group). RETr: Retention with reliable cursor. TM: Motor transfer. TVS: Visuospatial transfer. R: Group with reliable cursor; U: Group with unreliable cursor; RU: Group with reliable then unreliable cursor; UR: Group with unreliable then reliable cursor. Error bars denote standard error. Contrast analyses indicated grouping 1 > grouping 2 (*p* < .001) and grouping 3 > grouping 4 (*p* < .001).

Fig 3 depicts MT per element in each of the three phases of the experiment (i.e., acquisition, retention, and transfer) for each of the four groups (*i*.*e*., R, U, UR, and RU). For the acquisition phase (i.e., R1-R20), descriptive statistics indicated similar patterns of performance according to type of cursor, with longer MT when the cursor was unreliable (i.e., for U, for UR for the first 10 blocks, and for RU for the last 10 blocks) than when it was reliable (i.e., for R, for RU for the first 10 blocks, and for UR for the last 10 blocks). For the retention phase, especially for RETr, when the cursor was reliable for all four groups, we observed longer MT for the U group than for the UR, RU and R groups. Finally, differences in performance between the groups on the two transfer blocks indicated that a reliable cursor at the start of the acquisition phase (i.e., R and RU groups) produced a positive effect on MT in TVS, even if reliability was withdrawn later (i.e., RU). For TM, it was precisely the presence of a reliable cursor at the start of acquisition and its subsequent removal that had a positive impact on the MT. These observations were statistically tested with repeated-measures ANOVAs and planned comparisons on MT means.

### Effects of cursor reliability and switching on performance

We tested changes across the blocks according to the cursor reliability by comparing MT in a 4 (*Group*: R, U, RU, UR) x 20 (*Block*: R1-R20) two-way mixed ANOVA. This revealed main effects of *Group*, *F*(3,51) = 57.24, *p* < .001, η^2^ = 0.37, and *Block*, *F*(6.00,306.17) = 16.23, *p* < .001, η^2^ = 0.03, and a *Block* x *Group* interaction, *F*(18.01,306.17) = 66.19, *p <* .001, η^2^ = 0.38. As we expected to observe a major impact of the unreliable cursor on practice, we ran two separate planned comparisons that took account of the change of cursor for UR and RU. For the first 10 blocks, planned comparisons indicated significantly longer MT for U and UR (see Fig 3; grouping 1) than for R and RU (see Fig 3; grouping 2), β = 4888.37, *t*(56) = 10.90, *p* < .001. For Blocks R11-R20, results indicated significantly longer MT for U and RU (see Fig 3; grouping 3) than for R and UR (see Fig 3, grouping 4), β = 877.65, *t*(56) = 10.88, *p* < .001.

### Sequence-specific learning

**Fig 4:**
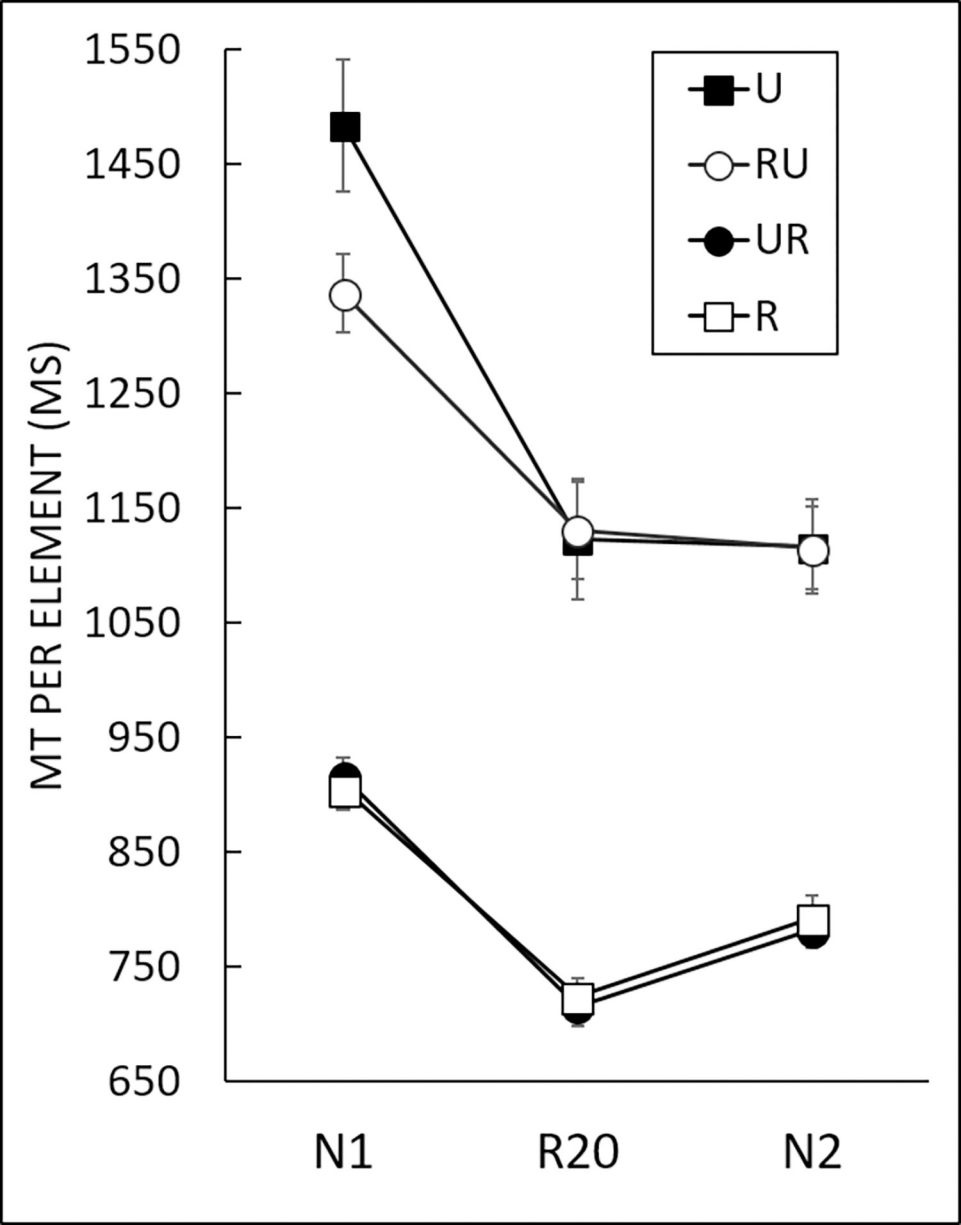
Movement time (MT) per element for each of the four groups for sequence-specific learning. N1 and N2: New blocks; R20: Repeated block. For these three blocks: Reliable cursor for R and UR, and unreliable cursor for U and RU. Error bars denote standard error. Planned comparisons between blocks indicated N2 < N1 (*p* < .001), R20 < N2 (*p* < .05), R20 < N1 (*p* < .001). Planned comparisons between groups indicated U and RU > R and UR for each of the three blocks (*p* < .001 for each comparison).

To assess sequence-specific learning of the repeated sequence, we compared the mean MT for the last repeated block (R20) and the pretest (N1) and posttest (N2) blocks [40] in a 4 (*Group*: R, U, RU, UR) x 3 (*Block*: N1, R20, N2) two-way mixed ANOVA (Fig 4). Analysis revealed main effects of both *Group*, *F*(3,59) = 95.88, *p* < .001, η^2^ = 0.58, and *Block*, *F*(1.48,87.18) = 97.92, *p* < .001, η^2^ = 0.17, and a Block x Group interaction, *F*(4.43,87.18) = 4.19, *p* < .001, η^2^ = 0.02. A planned comparison between MT for N2 and N1 was significant, β = 833.63, *t*(118) = 10.91, *p* < .001, indicating that the reduction in MT was partly due to practice per se. More importantly, the planned comparison between the repeated sequence after practice (R20) with the unpracticed sequence (N2) revealed a shorter movement time for R20 than for N2 across all groups, β = -162.76, *t*(118) = -2.131, *p* < .05. This reduction in MT was therefore also due to specific learning of the repeated sequence. We then assessed the effect of cursor reliability during the acquisition phase through a planned comparison between the four groups. A MT comparison on the most relevant repeated block (i.e., R20) indicated longer MT for U and RU than for R and UR, β = 755.71, *t*(149) = 11.66, *p* < .001. Therefore, the unreliable cursor had a detrimental effect on MT, but order of presentation (UR or RU) did not change this conclusion. The latter was confirmed by the results of planned comparisons on N1, β = 991.33, *t*(149) = 15.30, *p* < .001, and N2, β = 663.77, *t*(149) = 10.25, *p* < .001 (see Fig 4).

### Long-term learning

**Fig 5:**
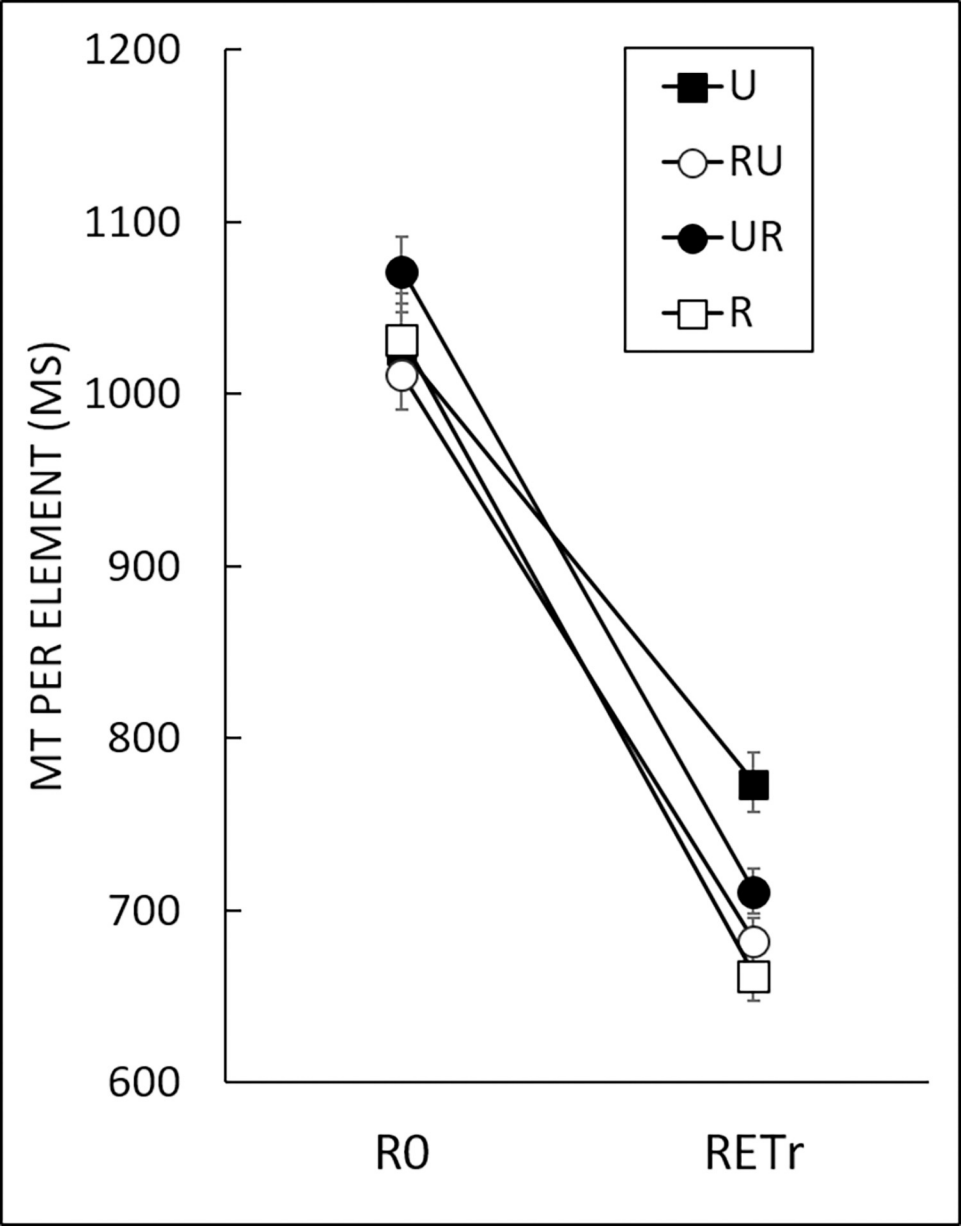
Movement time (MT) per element for each of the four groups for learning effect. R0: Repeated block; RETr: Retention with reliable cursor. For these blocks, all groups had the reliable cursor. Error bars denote standard error. A planned comparison between blocks indicated R0 > RETr (*p* < .001). A planned comparison between groups indicated U > other groups for RETr (*p* < .001).

The long-term effects of reliability and order of presentation (reliable or unreliable cursor first) on MT were analyzed with a 4 (*Group*: R, U, RU, UR) x 2 (*Block*; R0, RETr) two-way mixed ANOVA (Fig 5). These two blocks were performed with the reliable cursor for all groups. Analysis indicated main effects of both *Group*, *F*(3,55) = 4.94, *p* < .01, η^2^ = 0.03, *Block*, *F*(3,55) = 1206.06, *p* < .001, η^2^ = 0.81, as well as a Group x Block interaction, *F*(3,55) = 4.58, *p* < .01, η^2^ = 0.01. As illustrated in Fig 5, a planned comparison indicated that MT were longer at the start of the acquisition phase (i.e., R0) than in the retention phase (i.e., RETr), β = 1296.17, *t*(55) = 34.73, *p* < .001. More interestingly, for RETr, the planned comparison between U and the other groups was significant, β = 833.63, *t*(118) = 10.91, *p* < .001. This result indicated that a total absence of reliable online visual feedback during acquisition was detrimental to learning.

### Sensorimotor coding

**Fig 6:**
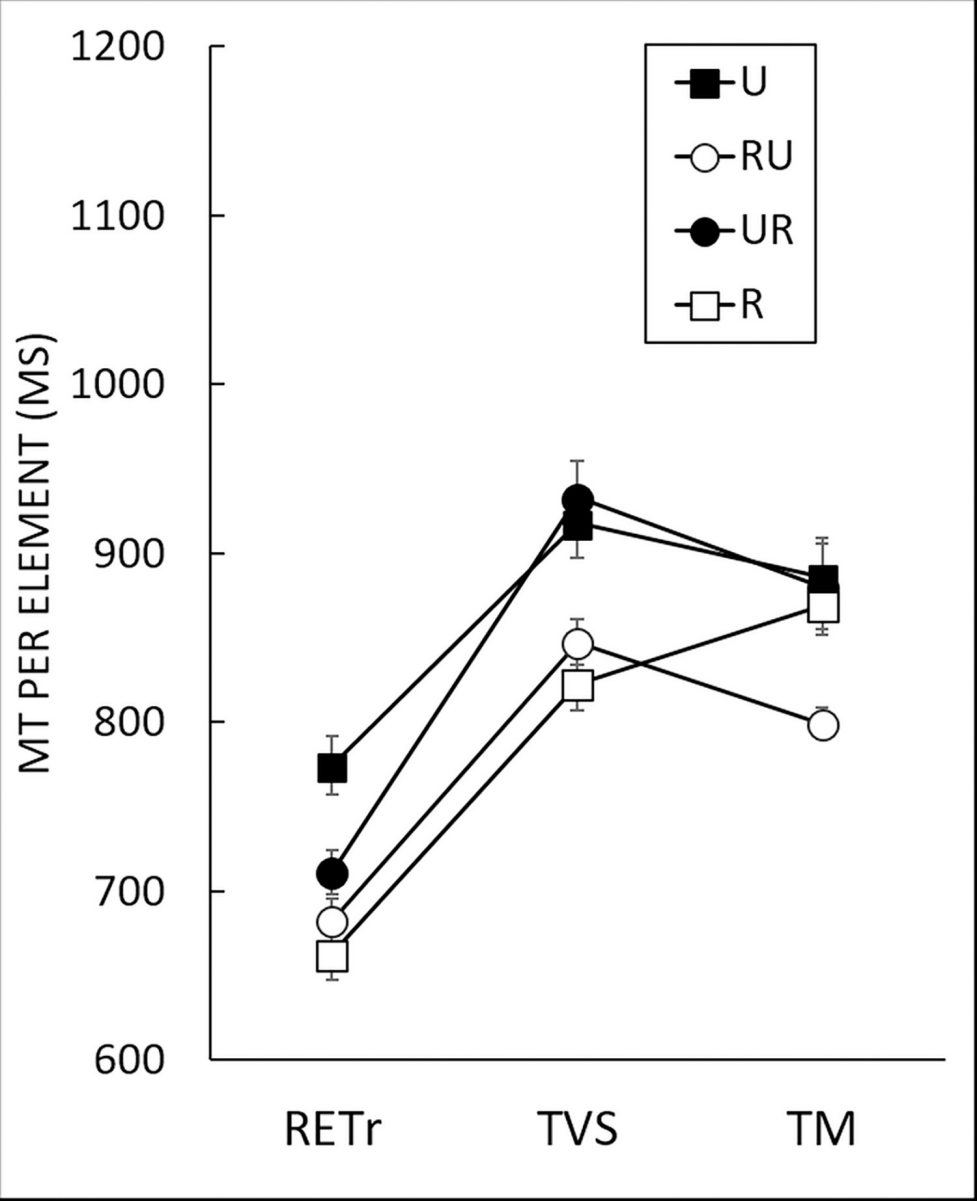
Movement time (MT) per element for each of the four groups for retention and transfer. RETr: Retention with reliable cursor; TVS: Visuospatial transfer; TM: Motor transfer. For these blocks, all groups had the reliable cursor. TVS was performed with the left hand and the repeated sequence. TM was performed with the left hand and the mirror sequence (see Fig 1). Error bars denote standard error. Planned comparisons between blocks indicate that RETr < transfers (TVS and TM, p < .001); TM < TVS for UR (p < .01) and RU (p < .05), and TM > TVS for R (p < .01). Planned comparisons between groups indicated U and UR > RU and R for TVS (p < .001), and R < the others for TM (p < .001).

We compared mean MT on retention and transfer (i.e., visuospatial and motor) to assess the persistence of the improvement in performance and to determine the nature of the coding. We conducted a 4 (*Group*: R, U, UR, RU) x 3 (*Block*: RETr, TVS, TM) two-way mixed ANOVA (Fig 6). The retention block was used to compare the efficiency of coding versus optimum performance [6,7,18,19]. Analysis indicated main effects of both *Group*, *F*(3,57) = 9.63, *p* < .001, η^2^ = 0.13, and *Block F*(1.82,103.82) = 240.11, *p* < .001, η^2^ = 0.48, and a Group x Block interaction, *F*(5.46,103.82) = 5.41, *p* < .001, η^2^ = 0.03.

Planned comparisons were conducted to understand this interaction and to characterize the coding. The planned comparison between the transfer (i.e., TVS and TM) and retention phases indicated shorter MT in RETr than in TVS and TM β = -1305.79, *t*(114) = -21.81; *p* < .001. We investigated the effects of reliability and order of presentation on visuospatial and motor transfers with two planned comparisons between groups. The first comparison indicated lower MT for R and RU than for U and UR, β = -214.47; *t*(108) = -5.95; *p* < .001. Therefore, a reliable cursor at the start of the acquisition phase (i.e., R and RU) had a positive effect on MT for the visuospatial transfer (TVS), even if reliability was withdrawn later (i.e., RU). For motor transfer (TM), it was precisely the presence of the reliable cursor at the start of acquisition and its subsequent removal that had a positive impact on the TM as revealed by the planned comparison, which indicated shorter MT for RU than for all the other groups, β = -245.19, *t*(108) = -4.00, *p* < .001 (see Fig 6).

We then assessed differences in transfer ability within groups. A planned comparison for R indicated shorter MT for TVS than for TM, β = -49.47, *t*(114) = -2.85, *p* > .05, as previously observed [7,18,19]. Studies have shown that exposure to an unreliable cursor enhances the development of motor coding [9,20] by reducing visual dominance, and the results of our comparisons revealed shorter MT for TM than TVS for both RU, β = 37.08, *t*(114) = 2.21, *p* < .05, and UR, β = 52.60, *t*(114) = 3.23, *p* < .01, although we only observed a trend toward significance for U, β = 32.26, *t*(114) = 1.73, *p* = .09.

## Discussion

The main objective of our study was to characterize the benefits of learning a motor sequence under variable conditions where the online visual feedback was more or less reliable, particularly for sensorimotor coding. More specifically, we tested the impact of modulating the level of reliability of online visual feedback during acquisition on the retention and transfer of a continuous dynamic arm movement task. Thus, delayed retention and visuospatial transfer and motor transfer tests (i.e., 24 h later) made it possible to characterize the sensorimotor coding and sequence learning processes (see [12] for a review).

Our study indicated for the first time that varying the reliability of online visual feedback during the acquisition phase (i.e., as for our RU and UR groups) modifies task coding modalities (see results of TVS and TM analyses) without damaging long-term sequence learning (see results for RETr). We even observed that starting the acquisition with reliable online visual feedback, replaced halfway through by unreliable feedback (i.e., RU group) significantly promoted motor coding, compared with the other groups. We discuss (i) the differences in sequence learning between our groups, (ii) the coding patterns regarding uncertainty, and (iii) the specific advantage of an acquisition phase where reliable feedback is withdrawn midway through (i.e., RU).

First, our results indicated that the improvement in performance observed across all groups could not result solely from visuohaptic calibration, as MT was shorter for the last repeated sequence block than for the new sequence in the posttest (i.e., sequence-specific learning analysis). As well as familiarizing themselves with the task, our participants therefore learned the specific motor sequence (see [41] for a review, [18,40]). Although it was important to collect sequence-specific learning data, we above all wanted to know whether this improvement was maintained over time, to ensure that the sequence had been learned [6,7,18,40]. We found that participants who had only received unreliable feedback (i.e., U group) exhibited poor retention after a 24-hr interval (i.e., long-term learning analysis). The literature has often shown that the absence of visual information has a negative impact on learning performance (e.g., [2,25,43–45]), but to our knowledge, the impact of unreliable online visual feedback on the learning of sequential tasks has never been investigated. Noise and uncertainty hinder sensorimotor control processes [46,47], and recent studies have indicated that uncertainty also impairs the learning of discrete tasks [8,9,21,48]. When online visual feedback is too inaccurate, it is less well integrated and dependency on other sensory sources increases [9,49]. We can therefore surmise that in the absence of reliable online visual feedback during the acquisition phase, participants in the U group had to fall back on other forms of feedback (e.g., proprioceptive), and ceased to use visual feedback to perform the task even when it later became reliable (i.e., in retention phase). Such is the cognitive constraint exerted by unreliable feedback that it transforms sensorimotor representations of the task [50], possibly making it harder to go back to using the more appropriate form of feedback (i.e., visual).

One of the most important challenges identified in the sensorimotor learning literature is to understand how the central nervous system translates, codes, integrates, and uses sensory information to perform motor tasks (for reviews, see for example [12,51–53]). Interestingly, the groups that had received unreliable feedback during the acquisition phase for some (i.e., UR and RU) or all (i.e., U) of the time modified their coding patterns accordingly, as they performed better on TM than on TVS. The literature generally describes the opposite pattern (as observed in the R group), with better performance on TVS than on TM, and genuine difficulty achieving satisfactory MT performances (e.g., [7,18,19]). Moreover, most studies have confirmed a preference for visuospatial coding (e.g., [6,7,18,19]), and relative underuse of motor coding (e.g., [7,18,19]). This preference is problematic, as it leads to a form of dependence on visual feedback *(specificity of practice hypothesis*; [2]) that is often observed in visuomanual tasks (e.g., [25,54–57]). In the case of our UR, RU and U groups, unreliability led individuals to rely more on other sensory sources, thus promoting motor coding [9]. Each system of representations produces particular learning and transfer abilities, as it encodes information that is specific to it [15–17]. Whereas visuospatial coding is generally linked to allocentric representations, motor coding is associated with egocentric representations [58]. There are therefore several advantages of combining visuospatial and motor coding, as mixed coding reduces dependence on visual feedback [2], and feeds and maintains a larger repertoire of sensorimotor representations (see [52], for a review) that can allow learners to move from one type of coding to another, according to task constraints [59]. However, although we did see an inversion of the usual coding pattern in UR, RU and U (i.e., shorter MT for TM than for TVS), the differences between the three groups in terms of movement times on the transfer tests clearly show that it is not enough to use unreliable feedback to optimize coding: it also depends on *timing*.

The main purpose of the RU and UR conditions was to expose participants to the two degrees of reliability within the same acquisition phase, so that they were able to use both visual information (i.e., when the feedback was reliable) and proprioceptive information (i.e., when the feedback was unreliable) to perform the task. In the wake of Moxley’s groundbreaking research [60], many studies have shown that variability is essential to optimize learning (for reviews, see [10,35]), as it influences information processing and decision-making processes concerning the task in hand [10]. As they learn, individuals make connections between the parameters of the assignment and the outcomes [31,61,62]. Thus, the more parameters they test, the broader their motor schema [32,63]. However, the most important and interesting finding of our study is that unreliability was beneficial, especially when it occurred at a later stage of the learning process. For participants in the RU group, who started with reliable feedback and continued with unreliable feedback, the MT for TVS was similar to that of their counterparts in the R group, while the MT for TM was actually shorter. According to Hikosaka and colleagues [3,4], the spatial locations of the effector and the stimuli are coded in visuospatial coordinates, and the motor patterns (i.e., muscle activations and joint configurations) are coded in motor coordinates. Their model specifies that even if the two representations emerge simultaneously and independently, visuospatial coding is generally dominant at the beginning of practice, with motor coding developing later. The variability encountered by the RU group was therefore congruent with this model. By providing accurate online visual feedback at the start of the acquisition phase, we promoted visuospatial coding. By introducing unreliability later on, we led participants to switch to other available types of information, particularly proprioceptive information, when it was most conducive to motor coding. It is also worth noting that visuospatial coding is described as explicit (i.e., humans consciously use relevant visual information such as the positions of their limbs and the stimuli) and effector-independent (i.e., not specific to an effector), whereas motor coding is described as implicit and specific to the effector producing the movement [3,4,63]. Accordingly, a reliable cursor at the start of the acquisition, followed by an unreliable cursor, produced a positive effect because it constituted a shift from the simplest (i.e., conscious effort, permutation of effector limbs without consequence, and therefore great adaptability) to the most complex (i.e., recourse to unconscious system, specification and parameterization of the task for a particular limb; [23]). Several theoretical perspectives presented earlier have proposed that independent codes, representations, or processing modules contribute to sequence learning. Further, these perspectives often argue that the development and reliance on these codes change over practice. For example, Hikosaka et al. [3], proposed that the reliance on codes shift from visual–spatial to motor over practice. Conversely, the present data are consistent with the notion that learning is determined by multiple codes that could be activated depending of the context and stage of learning. This multiple codes perspective has already be put forward by Kovacs et al. [7] by varying the amount of practice (from 1, 4 or 12 days). Our results indicate that both the reliability of visual information and the variability of practice may also contribute to the development of multiple coding.

## Conclusion

Our study assessed the impact of online visual feedback reliability on motor learning, focusing on the switch from reliability to unreliability during the acquisition phase. This specific condition promoted motor coding, which is seldom observed [7], without damaging long-term sequence learning. Thus, to optimize learning, the variability of online visual feedback must be adapted to the natural processing of representations [4], facilitating the processing of visual information first, then proprioceptive information. However, as recently underlined by Winter et al. [64], the role of proprioceptive information continues to be neglected, or is studied only indirectly. Future research should therefore focus on what happens to the learning of a motor sequence when the reliability of online proprioceptive feedback is manipulated.
